# Retraction Note: Genome assembly and annotation of *Meloidogyne enterolobii*, an emerging parthenogenetic root-knot nematode

**DOI:** 10.1038/s41597-025-04446-6

**Published:** 2025-01-30

**Authors:** Georgios D. Koutsovoulos, Marine Poullet, Abdelnaser Elashry, Djampa K. L. Kozlowski, Erika Sallet, Martine Da Rocha, Laetitia Perfus-Barbeoch, Cristina Martin-Jimenez, Juerg Ernst Frey, Christian H. Ahrens, Sebastian Kiewnick, Etienne G. J. Danchin

**Affiliations:** 1 Université Côte d’Azur, INRAE, CNRS, Institut Sophia Agrobiotech, Sophia Antipolis, France; 2 Strube Research GmbH & Co. KG, Hauptstraße 1, 38387 Söllingen, Germany; 3 Laboratoire des Interactions Plantes-Microorganismes, INRAE, CNRS, 31326 Castanet-Tolosan, France; 4 Agroscope, Molecular Diagnostics, Genomics & Bioinformatics, Wädenswil, Switzerland; 5 Swiss Institute of Bioinformatics, Bioinformatics, Wädenswil, Switzerland; 6 Julius Kühn Institute (JKI) - Federal Research Centre for Cultivated Plants Federal Research Centre for cultivated Plants, Messeweg 11/12, 38104 Braunschweig, Germany

Retraction Note to: *Scientific Data* 10.1038/s41597-020-00666-0, published online 05 October 2020

The authors have retracted this article after finding contamination of the underlying sequencing data with another species. A re-examination of the data suggests that the generated genome assembly is chimeric, corresponding to Meloidogyne incognita and Meloidogyne enterolobii. The underlying sequencing files also contained data from both organisms. Corrections resulted in the generation of new sequencing data and assembly, which have been peer reviewed and published at 10.1038/s41597-025-04434-w. This has rendered the validation of the dataset published in this earlier article to be invalid, with users encouraged to use 10.1038/s41597-025-04434-w instead, rather than the accessions listed in this retracted article.

Georgios D. Koutsovoulos, Marine Poullet, Juerg Ernst Frey, Christian H. Ahrens, Sebastian Kiewnick and Etienne G. J. Danchin agree with this retraction. Abdelnaser Elashry, Djampa K.L. Kozlowski, Erika Sallet, Martine Da Rocha, Laetitia Perfus-Barbeoch and Cristina Martin-Jimenez have not responded to correspondence from the Editor about this retraction.

